# Corneal hysteresis and intraocular pressure are altered in silicone-hydrogel soft contact lenses wearers

**DOI:** 10.1007/s10792-022-02270-0

**Published:** 2022-03-30

**Authors:** María Concepción Marcellán, Laura Remón, Francisco J. Ávila

**Affiliations:** Departamento de Física Aplicada, Universidad de Zaragoza, C/ Pedro Cerbuna, 12, 50009 Zaragoza, Spain

**Keywords:** Corneal biomechanics, Soft contact lenses, Intraocular pressure, Glaucoma

## Abstract

**Purpose:**

The aim of this work is to determinate the effects in the physical parameters in terms of intraocular pressure (IOP) and central corneal thickness (CCT) and corneal biomechanics in terms of corneal resistance factor (CRF) and corneal hysteresis (CH) of wearing silicone-hydrogel soft contact lenses (SiH-CLs) in young adult subjects during a short-term follow-up.

**Methods:**

40 eyes of 20 healthy patients with a mean age of 22.87 ± 4.14 were involved in this study. Subjects with corneal diseases, dry eye, irregular astigmatism or who have been previous contact lens wearers were excluded. The ocular response analyzer (Reichert Ophthalmic Instruments) was used to measure CH, CRF and IOP and Scheimpflug imaging (the GALILEI™ Dual Scheimpflug camera analyzer, Ziemer) was used to measure CCT before and 10 days (Group 1) and 20 days (Group 2) after wearing the SiH-CLs.

**Results:**

IOP was significantly decreased 10 days after using the SiH-CLs (*p* = 0.009). Within the 20 days' period, Group 2 revealed an even more pronounced decrease in IOP (*p* = 0.003) while CH increased significantly (*p* = 0.04). CCT and CRF did not show a significant change during the period of SiH-CLs use. Our finding allowed obtaining an empirical expression that relates IOP, CCT, CRF and CH within a biomechanical compensation experimental model.

**Conclusions:**

Corneal biomechanical parameters and physical properties of the cornea may be altered due to SiH-CLs use. Our findings could have an impact on the management of glaucoma progression and ocular hypertension.

## Introduction

The biomechanical properties of the cornea are responsible of its stability and functionality with high impact in vision [1]. The cornea is characterized by elastic and viscoelastic properties [2] that play a fundamental role in refractive surgery [3], orthokeratology [4], keratoconus progression [5], glaucoma or corneal ectasia [6].

Refractive errors are very common human eye disorders and are leading causes of visual impairment worldwide. Soft contact lenses (CLs) are non-surgical vision correction alternatives to spectacles, with more than 140 million wearer’s worldwide [7]. The developments in CLs design technologies have focused on comfortability, tear film stability, biocompatibility and the development of new materials [8]. On the one hand, hydrogel CLs offer materials with good wettability, optical quality and comfortability. However, the oxygen permeability is moderate and this can lead to symptoms of dryness at the end of the day.

Oxygen-deficient metabolism (i.e., hypoxia) leads to metabolic changes and then to alterations in corneal biomechanics [9]. In this sense, silicone hydrogel CLs (SiH-CLs) have been chosen as the best option by new CLs users in the last decades [10]. This type of material provides more than five times oxygen permeability through the material compared to standard hydrogel materials [11], preventing the hypoxia-related [12] physiological changes within the corneal tissue and allowing patients wearing CLs for long daily use.

Despite the advantages of SiH-CLs, SiH materials present higher modulus of elasticity than conventional hydrogel lenses. Although the new generation of SiH-CLs have balanced the oxygen transmissibility obtaining a lower modulus of elasticity compared to the first generation, these two factors continue playing an important role in corneal biomechanics [13].

Some studies have reported topographic changes in both central and peripheral cornea [14, 15] after short-term CLs wearing including corneal swelling [16], optical aberrations [17] or loss of radial symmetry [18]. Those physical changes were observed in both soft hydrogel [19] and silicone hydrogel CLs [16].

In this sense, previous studies reported alterations in corneal biomechanics induced by long-term soft CLs wearing [20]. Lu et al. [21], reported alterations in the biomechanical properties of the cornea after wearing soft contact lenses during eye closure. Matalia et al. [22], demonstrated correlation between corneal stiffness and myopia. Few studies have investigated biomechanical changes after short-term period of wearing contact lenses [15, 23]. Tyagi et al. [15] reported changes in corneal thickness and morphology after analyzing different types of soft toric contact lenses materials (silicone hydrogel and hydrogel). Radaie-Moghadam et al. [23] studied the effect of toric soft contact lens wear on corneal biomechanical properties over a 3-month period.

The knowledge about any physical and/or biomechanical change in the short-term use of CLs could be crucial in occasional wearers, patients with corneal pathologies such as keratoconus, ectasia or glaucoma, in order to detect early contraindications or discard candidates for whom some transient biomechanical alterations may compromise the corneal stability.

In this sense, air-puff tonometers provide excellent measurement of corneal biomechanics [24] and have been successfully employed to analyze corneal alterations in glaucoma patients [25], keratoconus degeneration [26], or patients undergoing refractive surgery.

The aim of our work is to carry out a prospective observational study to determine the impact of wearing SiH-CLs on biomechanical and corneal physical parameters during 10 and 20 days of follow-up periods by combining non-contact air-puff tonometry and Scheimpflug imaging.

## Methods

### Participants

This research was reviewed by an independent ethical review board and conforms to the principles and applicable guidelines for the protection of human subjects in biomedical research (Ethical Committee of Research of the Health Sciences Institute of Aragon, Spain) approved with reference: C.P.-C.I.PI20/377. Measurements procedure and data collection were carried out according to the tenets of the Declaration of Helsinki. All participants were informed about the nature, risks and possible adverse consequences of the study and signed an informed consent document. The participants were European Caucasian population, non-wearers contact lenses students from the school of Optics and Optometry of the University of Zaragoza (Spain), a total of 40 eyes from 20 healthy young adult subjects (mean age of 22.87 ± 4.14 years old) were involved in the study.

Inclusion criteria consisted of age range between 18 and 28 years with myopia, hyperopia or astigmatism and best corrected visual acuity at distance at least of 20/20 for each eye. Exclusion criteria were history of using any type of contact lenses, corneal diseases or surgery, dry eye, or irregular astigmatism. In addition, patients who presented contact lens intolerance during the first day of use were excluded.

### Contact lens use

The short-term wear time was divided into two different temporal intervals: 10 participants were asked to wear SiH-CLs 8 h of uninterrupted daily use during 10 days, and the second group (10 participants) during 20 days with the same conditions.

Silicon hydrogel soft CLs of monthly replacement (Horizont Bio, Tiedra Farmacéutica S.L. Spain) were selected for this study. Table 1 shows the technical specifications:

**Table 1:** Contact lenses technical specifications

Material	Fanfilcon A
Hydration	55%
Central Thickness	0.08 mm
Dk/t	110
Modulus	0.6 Mpa
Optical design	Aspheric
Border design	Round shape
UV filter	Class 1

Keratometric, pachymetric parameters and ocular refractive errors were measured using a dual Scheimpflug analyzer (see “Experimental Procedure” for details) and an open-view autorefractometer (Grand Seiko, WAM-5500), respectively. Subjective refraction and evaluation of the anterior segment of the eye were conducted by an experienced optometrist. Total diameter and the effective optic zone radius of the CLs were chosen following the manufacturer’s guidelines.

### Experimental procedure and data analysis

Ocular response analyzer (ORA, Reichert Instruments, Depew, NY, USA) is a non-contact air puff applanation tonometer that provides corneal hysteresis (CH) and corneal resistance factor (CRF) measurements. Briefly, CH can be defined as the energy dissipation when an external stress is applied resulting in a time-dependent stain unlike purely elastic materials, that immediately recover the initial state once the stress stops. Thus, CH is a function of the corneal viscoelastic behavior.

CRF is related to the pure elastic properties of the cornea [27]. Therefore, CH and CRF are representative parameters of the biomechanical properties of the cornea. ORA instrument also provides compensated IOP measurements independent of corneal biomechanics and pachymetry.

In our study we first measured the central corneal thickness (CCT) using a dual Placido-Scheimpflug imaging analyzer (the GALILEI™ Dual Scheimpflug camera analyzer, Ziemer). Then, CCT measurement was incorporated to the operating interface of the ORA´s operating interface to start biomechanical measurements.

The measurement protocol was a simple sequential procedure: first Scheimpflug analyzer measures the CCT of the participant and next after incorporating this parameter to ORA instrument the compensated IOP (hereinafter IOP) CH and CRF are measured by air-puff applanation. Each final data were the average of three sequential experimental measurements.

The measurements were separated in two temporal groups: 10 and 20 days after SiH-CLs wearing. The control measurements were acquired after the first insertion of the CLs, the follow-up measurements were carried out two hours after asking the participants to remove the CLs once the wearing time ended. The reason for waiting two hours between contact lens removal and the measurements was motivated by the reported fluctuation [21] in corneal thickness after CL removal that recovers baseline values after 100 min.

Collected data were stored into an Excel spreadsheet. Every participant was identified by a reference number, no personal data were included. Once the experiment was finished, the spreadsheet was migrated to Origin Lab software (Origin Lab Corp.) for graphical representations and data analysis. The statistical analysis was performed using the advanced statistical tool including two-sample hypothesis tests and Spearman Rank Correlation Coefficient.

## Results

Intrinsic biomechanical (CRF and CH) and bio-physical (IOP and CCT) parameters were measured in a total of 40 eyes of 20 young healthy participants before and after wearing SiH-CLs. Two temporary periods of SiH-CLs use were considered in this study: 10 and 20 days, for which the described measurements were acquired immediately before and after starting and ending wearing periods.

### Follow-up of biomechanical and physical parameters after SiH-CLs wearing

**Fig. 1:**
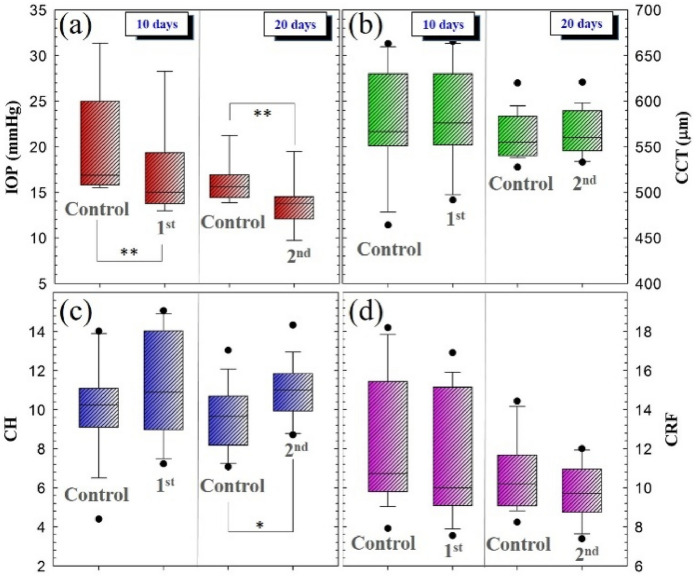
Box diagrams of IOP (**a**), CCT (**b**), CH (**c**) and CRF (**d**) values comparing control measurements with each temporal group. For each box, the median (central line inside each box), the Q1 and Q4 quartiles (lower and higher borders of each box, respectively) and maximum and minimum values (whiskers) are shown. (*p < 0.05; **p < 0.005)

Figure 1 compares the IOP (Fig. 1a), CCT (Fig. 1b), CH (Fig. 1c) and CRF (Fig. 1d) values of the two groups of participants. The mean IOP showed a significant reduction after 10 (*p* = 0.009) and 20 days (*p* = 0.003) of SiH-CLs use. The pachymetry revealed a mean increase in CCT, although no significant differences were found, a slight edema was observed in the first group (*p* = 0.35) which reverted to stabilization in the 20-days group (*p* = 0.48), recovering the control values.

In addition, the analysis of the biomechanical parameters revealed how the use of SiH-CLs leaded to an increase in CH in both groups, showing a statistical shift in the 20-days group (*p* = 0.04). On the other hand, the use of SiH-CLs did not significantly affect the CRF, however the behavior of this parameter (Fig. 1d) showed the opposite trend as observed in CCT (Fig. 1b), that is, a more accused decrease after 20 days of SiH-CLs wear (*p* = 0.13) compared to the 10-days group (*p* = 0.32).

**Fig. 2:**
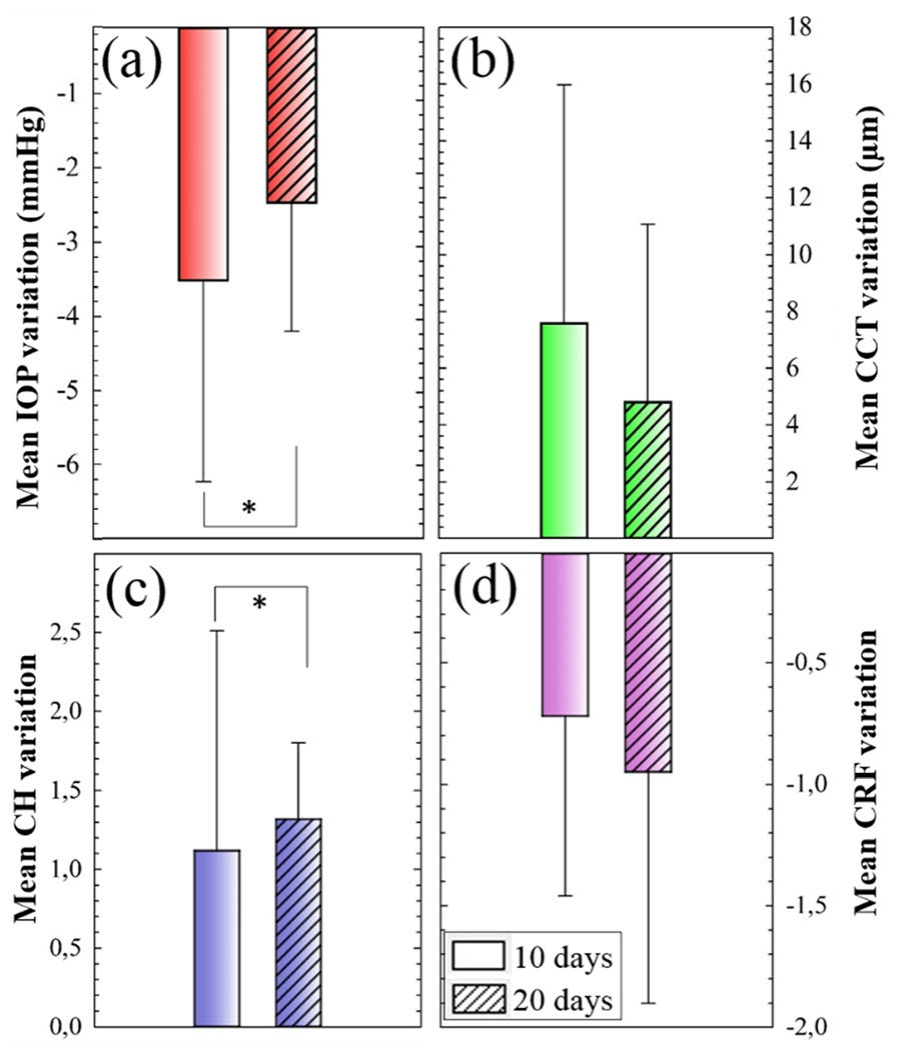
Mean variation PIO (**a**), CCT (**b**), CH (**c**) and CRF (**d**) values of the two studied temporal groups (10 and 20 days). (*p < 0.05)

For the sense of completeness, Fig. 2 compares the mean variation values of the parameters plotted in Fig. 1. This mean variation is calculated as the difference between the averaged values after SiH-CLs wearing and the control measurements. The bio-physical and biomechanical parameters that presented significant alterations as a consequence of SiH-CLs wearing were IOP (*p* = 0.008) and CH (*p* = 0.04), respectively.

In that sense, the increase in the time of SiH-CLs wearing implied a reduction in the variation in the IOP value and an increase in CH. Whereas CCT and CRF were not greatly affected by the use of SiH-CLs, those parameters showed opposite mean variations according to the behaviors observed in Fig. 1. That is, as the wearing time increases, the induced corneal edema is less pronounced while CRF shows higher variation in average rate.

### Relationship between physical and biomechanical parameters

The results presented in Figs. 1 and 2 assessed the corneal alterations as a consequence of wearing SiH-CLs during two short follow-up periods measured by means of biomechanical (CH and CRF) and pure physical parameters (CCT and IOP). These alterations were quantified by measuring CH and CRF parameters. Nevertheless, for a better understanding on how the biophysics of the cornea is modified, it is necessary to investigate how the different properties are related.

A Spearman´s correlation analysis was performed by including the whole dataset (i.e., including both control and the two temporal groups) of the experiment. The statistical analysis revealed significant relationships between the biomechanical parameters and between physical and biomechanical data.

**Fig. 3:**
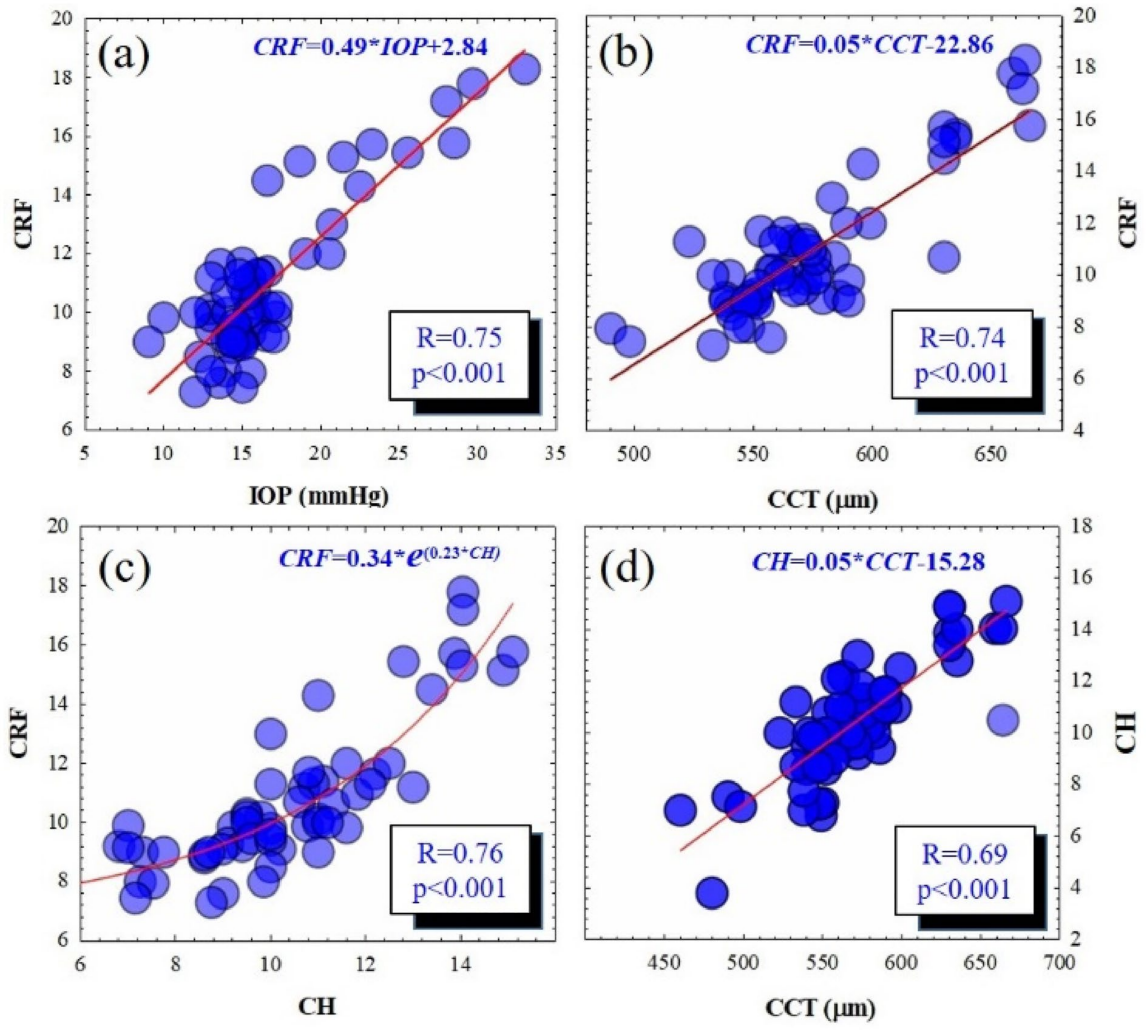
Correlation and best mathematical fitting between physical and biomechanical parameters. The correlation results are shown within the shaded boxes

Panels (a) and (b) of Fig. 3 shows linear correlations between physical parameters (IOP and CCT) and the CRF parameter. In both cases, higher corneal resistance is related to higher IOP and corneal thickness with statistical significance (the results of the correlation are shown in the shaded boxes). CRF, IOP and CCT parameters are related by means of the following experimental fittings:

1 $$ {\text{CRF}} = 0.49*{\text{IOP}} + 2.84 $$

2 $$ {\text{CRF}} = 0.05*{\text{CCT}} - 22.86 $$

Figure 3d also relates the corneal thickness (CCT) and the viscoelastic property (CH) that according to the results, the greater the corneal pachymetry, the higher the viscoelasticity or the viscoelastic response of the corneal tissue under an external stress.

From data of Fig. 3c, the relationship between viscoelastic (CH) and elastic (CRF) properties can be analytically computed from Eq. (3):

3 $$ {\text{CRF}} = 0.34 \cdot e^{{023 \cdot {\text{CH}}}} $$

The best mathematical fitting was an exponential function. This nonlinear behavior of the CRF as a function of the hysteresis can be explained by the transient viscous response of the cornea. This finding plays an important role in this study to explain one of the compensation mechanism acting as biomechanical response when the intraocular pressure fluctuates but the corneal thickness and resistance remain constants.

In this sense, biomechanical and physical corneal parameters that can be unified through an empirical biophysical expression by adding Eqs. (1), (2) and (3) as follow:

4 $$ {\text{CRF}} = \frac{1}{3} \cdot \left( {0.49 \cdot {\text{IOP}} + 0.05 \cdot {\text{CCT}} + 0.34 \cdot e^{{023 \cdot {\text{CH}}}} - 22.86} \right) $$

From a dynamical point of view, Eq. (4) describes a biomechanical compensation model to explain how the viscoelastic response compensates for IOP and CCT imbalances, in order to preserve the corneal resistance.

## Discussions and conclusions

Corneal swelling, shape alterations, refractive stability or induced optical aberrations are crucial factors for normal vision [1] that must also be specially controlled in severe corneal pathologies such as keratoconus [5], ectasia [28] or other ocular degenerative diseases with corneal manifestations such as glaucoma, for instance.

In this sense, there has been an increasing effort to understand how corneal biomechanics is affected by soft contact lenses wearing. It is well-known that both hydrogel and silicone hydrogel soft contact lenses induce structural corneal alterations: thickness [14], warpage [29] or morphology [16].

Lau and Pye [30] reported biomechanical overestimation measured with applanation tonometry due to the temporal hydration or induced corneal edema as a consequence of wearing hydrogel soft contact lenses. This overestimation is due to an increasing in corneal stiffness induced by corneal hyper-hydration.

In addition, Lu and coworkers studied biomechanical changes after inducing corneal edema by eye closure while wearing Hydroxyethyl-methacrylate soft CLs [21] during 3 h. Immediately after lens removal they found significant correlation between IOP and CRF with corneal thickness but no association of CH with corneal edema. Both CRF and CCT recovered the baseline values after 100 min. These two studies revealed short-term time-dependent changes in CRF and CCT because of the induced corneal edema.

In this work we have investigated the corneal biomechanics, IOP and CCT alterations after wearing SiH-CLs during two temporal groups: 10 and 20 days. Results herein showed that after 10 days the IOP is significantly reduced, whereas CH slightly increases and CRF and CCT remain without important variations.

The longer period of follow-up (20 days) revealed a stronger decrease in IOP and a statistical increase in CH. No induced edema or increased corneal stiffness (CRF) were observed. This inverse relationship can be explained by the La Place´s law: as the IOP increases the rigidity is greater and therefore the viscous damping reduces, that is, IOP and CH are negatively correlated [31, 32].

In this sense, Radaie-Moghadam and collaborators [23] extended the following time of corneal biomechanics after using soft toric contact lenses up to three months. They found a significant reduction in both CH and CRF after 1 month of CLs wearing, but not a significant variation in CCT or corneal swelling. This last finding is consistent with our results, however the main discrepancies in our study and the study by Radaie-Monghadam et al. [23] can be due to an absence of IOP analysis that could explain an increase in CH instead of the observed reduction in our study. A second discrepant factor may lie in the relationship between corneal biomechanics stiffness and the refractive error [22] and the fact that all the subjects were chosen to present regular astigmatism. Other factors such as contact lens properties or patient´s age difference could explain the discrepancies.

Although it is clear the direct influence of soft CLs wear in corneal biomechanics, a deep knowledge on how biomechanical and physical corneal parameters are related is necessary. In our work we have analyzed the whole data set of the study (including control and the two temporal groups) in order to find the possible relationships between pure physical (IOP and CCT) and biomechanical corneal parameters (CH and CRF).

Our results revealed that IOP is positively correlated to CRF, that is, the higher the elastic property the higher the measured IOP. CRF was also linearly associated with CCT, these finding are consistent with the results reported by Bhan et al. [33].

From a physiopathological point of view, an increase in CCT has been associated to ocular hypertension [34–36] what could explain the observed increase in CRF while measuring the IOP according to the results previously reported by Sha et al. [37]. They observed highest values of CRF (and therefore corneal rigidity) in ocular hypertension eyes. More recently, the positive correlation between corneal thickness and stiffness was demonstrated using optical coherence elastography [38].

In 2009, Mangouritsas et al. [39] reported a strong association between CH and CCT in healthy eyes that becomes weaker as IOP increases in glaucomatous eyes. Our results are in good agreement with this study as revealed a positive correlation between CH and CCT (Fig. 3d).

Finally, the exponential relationship found between CH and CRF allowed to obtain an analytical biophysical expression that unifies IOP, CH, CRF and CCF parameters. This equation allows explaining the follow-up biomechanics after SiH-CLs wearing represented in Fig. 1 as follow:

If the IOP decreases while CCT remains without significant fluctuations, in order to preserve the corneal resistance the viscoelastic response increase for mechanical compensation. These findings suggest that while the elastic property is weakening, the viscosity increases to compensate the corneal biomechanics and preserve ocular stability.

To conclude, unveiling the biomechanical properties of the human cornea is crucial for understanding the development of corneal diseases and new treatments. The main findings of our work lie in fact that the use of SiH-CL during a short-term period of wearing time reduces the IOP as the CH increases. Considering that CH has been proved a biomarker of visual field loss in glaucomatous eyes [40], future work of this study could help in the development of new biomechanical treatment against the progression of glaucoma.
